# Characteristics and Precipitating Circumstances of Suicide Among Children Aged 5 to 11 Years in the United States, 2013-2017

**DOI:** 10.1001/jamanetworkopen.2021.15683

**Published:** 2021-07-27

**Authors:** Donna A. Ruch, Kendra M. Heck, Arielle H. Sheftall, Cynthia A. Fontanella, Jack Stevens, Motao Zhu, Lisa M. Horowitz, John V. Campo, Jeffrey A. Bridge

**Affiliations:** 1 Center for Suicide Prevention and Research, Abigail Wexner Research Institute at Nationwide Children’s Hospital, Columbus; 2 Department of Pediatrics, The Ohio State University College of Medicine, Columbus; 3 The Ohio State University Wexner Medical Center Department of Psychiatry and Behavioral Health, Columbus; 4 National Institute of Mental Health, National Institutes of Health, Office of the Clinical Director, Bethesda, Maryland; 5 Johns Hopkins University School of Medicine, Department of Psychiatry and Behavioral Sciences, Baltimore, Maryland

## Abstract

**Importance:**

Suicide is the eighth leading cause of death among children aged 5 to 11 years, with rates increasing during the past decade. A better understanding of factors associated with childhood suicide can inform developmentally appropriate prevention strategies.

**Objective:**

To examine characteristics and precipitating circumstances of childhood suicide.

**Design, Setting, and Participants:**

This qualitative study examined restricted-use data from the National Violent Death Reporting System (NVDRS) regarding child suicide decedents aged 5 to 11 years in the US from 2013 to 2017. The NVDRS is a state-based surveillance system that collects data on suicide and violent deaths in 50 states, with restricted-use data available from 37 states. Details and context related to suicide deaths were identified through a content analysis of case narratives from coroner or medical examiner and law enforcement reports associated with each incident.

**Exposures:**

Characteristics and precipitating circumstances associated with suicide cited in the coroner, medical examiner, and law enforcement case narratives.

**Main Outcomes and Measures:**

Suicide incidence and risk factors for suicide including mental health, prior suicidal behavior, trauma, and peer, school, or family-related problems.

**Results:**

Analyses included 134 child decedents (101 [75.4%] males; 79 [59.0%] White individuals; 109 [81.3%] non-Hispanic individuals; mean [SD] age, 10.6 [0.8] years). Most suicides occurred in the child’s home (95.5% [n = 128]), and more specifically in the child’s bedroom. Suicide by hanging or suffocation (78.4% [n = 105]) was the most frequent method, followed by firearms (18.7% [n = 25]). Details on gun access were noted in 88.0% (n = 22) of suicides by firearm, and in every case, the child obtained a firearm stored unsafely in the home. Findings revealed childhood suicide was associated with numerous risk factors accumulated over time, and suggest a progression toward suicidal behavior, especially for youth with a history of psychopathology and suicidal behavior. An argument between the child and a family member and/or disciplinary action was often a precipitating circumstance of the suicide.

**Conclusions and Relevance:**

This qualitative study found that childhood suicide was associated with multiple risk factors and commonly preceded by a negative precipitating event. Potential prevention strategies include improvements in suicide risk assessment, family relations, and lethal means restriction, particularly safe firearm storage. Future research examining the myriad aspects of childhood suicide, including racial/ethnic and sex differences, is needed.

## Key Points

**Question:**

What characteristics and precipitating circumstances are associated with childhood suicide?

**Findings:**

In this multistate population-based qualitative study, childhood suicide was associated with multiple risk factors including mental health, prior suicidal behavior, trauma, and family or peer relation issues, with most suicides occurring by hanging or suffocation in the decedent’s bedroom. Firearms were the second most prevalent suicide method, and among cases with detailed information, all children obtained guns stored unsafely in the home.

**Meaning:**

The findings underscore the importance of early suicide prevention efforts that include improvements in suicide risk assessment, family relations, and lethal means restriction, particularly safe firearm storage.

## Teaser

This qualitative study assesses characteristics and precipitating circumstances of suicide among children aged 5 to 11 years in the US.

## Introduction

Youth suicide is a major public health concern. Although uncommon prior to adolescence, suicide was the eighth leading cause of death among children aged 5 to 11 years in the United States,^1^ and accounted for 2.3 deaths per 1 million youth in 2019.^2^ In a study examining US emergency department visits for youth with suicidal ideation and attempts, 43% of cases from 2007 to 2015 were in children aged 5 to 11 years.^3^ An additional analysis reported rates in this age group increased 14.7% annually between 2012 to 2017.^4^ Despite these troubling statistics, childhood suicide research has received limited attention compared with suicide in adolescents.

Evidence suggests suicide risks for adolescents such as psychopathology,^5,6,7^ family factors,^8,9,10^ and maltreatment^11,12^ are also associated with childhood suicide, but there are notable differences.^13,14,15^ In a study of youth aged 5 to 11 years and 12 to 18 years with a history of suicidal behavior, younger youth were more likely to experience bullying and have a family history of depression.^13^ An additional study found that suicide decedents aged 5 to 11 years were more likely to be male, Black, to die at home by hanging or strangulation, be diagnosed with attention-deficit/hyperactivity disorder (ADHD), and less likely to experience depression compared with decedents aged 12 to 14 years.^15^

In response to a call for action from the National Institute of Mental Health task force on child suicide research,^16^ the current study examines characteristics and precipitating circumstances of suicide in children using the National Violent Death Reporting System (NVDRS).^17^ The NVDRS is a state-based surveillance system that collects data on suicide and violent deaths.^17^ Information was obtained from coroner, medical examiner, and law enforcement reports associated with each death. Although previous research has examined quantitative data elements to characterize childhood suicide,^13,14,15^ this study’s qualitative approach uniquely captures additional details and context related to each incident. A better understanding of underlying factors associated with childhood suicide can inform developmentally appropriate prevention strategies for this population.

## Methods

This qualitative study analyzed NVDRS data on child suicide decedents between 2013 and 2017. At the time of the study, 50 US states participated in the NVDRS; however, restricted-use data were only available from 37 states.^17^ An earlier study on childhood suicide using NVDRS data was limited to data through 2012 for 17 states, whereas this study has provided a 5-year update with information from 37 states.^15,17^ A total of 136 suicides for children aged 5 to 11 years were identified for the study period. Incidents where the underlying cause of death was missing or not coded as suicide based on the *International Statistical Classification of Diseases and Related Health Problems, Tenth Revision (ICD-10)* (X60-X84, Y87.0, and U03)^2^ were reviewed by 3 authors (D.R., K.H., and J.B.). Of these 136 incidents, 2 were misclassified as suicide and were excluded, leaving 134 cases. Demographic information in the NVDRS including age, sex, and race/ethnicity was obtained from death certificate data. Race/ethnicity was assessed to identify potential disparities in suicide deaths, and categorized based on the Office of Management Standards for the Classification of Federal Data on Race and Ethnicity.^18^

This study was considered exempt according to review policies for deidentified data of The Abigail Wexner Research Institute at Nationwide Children’s Hospital institutional review board. Informed consent was not needed because the data were publicly available and deidentified. This study followed the Standards for Reporting Qualitative Research (SRQR) reporting guideline.^19^

### Variables and Analysis

Descriptive statistics were conducted for sex, age, race/ethnicity, method of suicide, and injury location. Methods associated with grounded theory and thematic analysis, including open coding procedures,^20,21^ were used to identify overarching themes describing the incident. Three authors (D.R., K.H., and J.B.) individually performed a content analysis of autopsy and law enforcement narratives to inductively identify characteristics and precipitating circumstances of suicide and group them into themes. Authors reflected an interdisciplinary team with backgrounds in epidemiology, public health, and clinical social work. A constant comparative approach was used to refine themes into more succinct categories.^20,21^ Discrepancies in coding were resolved and data saturation confirmed by reaching mutual consensus after discussion between authors. Statistical analyses were performed with SPSS, version 26.0 (IBM Corp).

## Results

Among the study’s 134 decedents, the sample was predominantly male (75.4% [n = 101]), White (59.0% [n = 79]), and non-Hispanic (81.3% [n = 109]); the mean (SD) age was 10.6 (0.8) years (Table 1). Most suicide deaths occurred in the child’s home (95.5% [n = 128]), and more specifically the child’s bedroom (65.6% [n = 84]) (Table 2). Children most often died by hanging or suffocation (78.4% [n = 105]) with a belt or other item of clothing; 18.7% of children (n = 25) died by firearm, and more than half (52% [n = 13]) involved a handgun. Details on gun access were noted in 88.0% of suicides (n = 22) by firearm. In each case, the child obtained a firearm stored unsafely in the home. In one example “the father kept his gun loaded in the front room where it was not stored securely” and in another “the victim's mother kept the pistol and ammunition unlocked in her nightstand.” Suicide by poisoning or other means occurred in 3.0% of deaths (n = 4).

**Table 1.** Individual Characteristics of Suicide Decedents Aged 5-11 Years in 37 US States and the District of Columbia, 2013-2017^a^

Demographics	Decedents, No. (%)
Age, y	
5-9	12 (9.0)
10-11	122 (91.0)
Mean (SD)	10.6 (0.8)
Sex	
Male	101 (75.4)
Female	33 (24.6)
Race	
White	79 (59.0)
Black	43 (32.1)
Other	12 (9.0)
Ethnicity	
Non-Hispanic	109 (81.3)
Hispanic	23 (17.2)
Other	2 (1.5)

Abbreviation: NVDRS, the National Violent Death Reporting System.

^a^ NVDRS participating states and years: Alaska, Maryland, Massachusetts, New Jersey, Oregon, South Carolina, and Virginia (2003-2017); Colorado, Georgia, North Carolina, Oklahoma, Rhode Island, and Wisconsin (2004-2017); Kentucky, New Mexico, and Utah (2005-2017); Ohio (2011-2017); Michigan (2014-2017); Arizona, Connecticut, Hawaii, Kansas, Maine, Minnesota, New Hampshire, New York, and Vermont (2015-2017); Illinois, Indiana, Iowa. Pennsylvania, and Washington (2016-2017); and California, Delaware, District of Columbia, Nevada, Puerto Rico, and West Virginia (2017).

**Table 2.** Suicide Method and Location of Injury From Autopsy and Law Enforcement Narratives of Suicide Decedents Aged 5-11 Years in 37 US States and the District of Columbia, 2013-2017

Suicide method	Decedents, No. (%)
Hanging or suffocation	105 (78.4)
Belt	38 (36.2)
Scarf	6 (5.7)
Shoelace	4 (3.8)
Bedsheet	4 (3.8)
Rope	6 (5.7)
Other/unknown	47 (44.8)
Firearm	25 (18.7)
Handgun	13 (52.0)
Rifle/shotgun/other larger firearm	5 (20.0)
Other/unknown	7 (28.0)
Poisoning/other^a^	4 (3.0)
Location of injury	
Decedent's residence	128 (95.5)
Child's bedroom	84 (65.6)
Closet	33 (39.3)
Bed	23 (27.4)
Other/unknown	28 (33.3)
Other bedroom	8 (6.3)
Bathroom	10 (7.8)
Basement or garage	6 (4.7)
Residence, other	20 (15.6)
School	1 (0.7)
Other/unknown	5 (3.7)

^a^ Other suicide methods included fall, transportation-related, drowning, cut/pierce, fire/burn, and unspecified methods.

Four themes emerged regarding precipitating circumstances including (1) mental health and suicide-related concerns, (2) trauma, (3) family-related problems, and (4) school or peer-related problems (Table 3). Thematic boundaries often overlapped because of the complexity associated with each decedent’s situation (Figure). For example, children with mental health concerns or a history of suicidal behavior often had traumatic histories related to adverse family situations. School problems frequently resulted in parent-child conflict and were more likely to occur in children with mental health concerns. Technology was intertwined across categories, either to communicate suicidal statements and death wishes, as a mechanism that exposed children to suicide, or related to disciplinary actions associated with school and behavioral problems.

**Table 3.** Themes and Categories of Precipitating Circumstances from Coroner, Medical Examiner, and Law Enforcement Narratives of Suicide Decedents Aged 5-11 Years in 37 US States and the District of Columbia, 2013-2017^a^

Theme	Cases, No. (%)
Mental health and suicide-related issues	
Mental health	37 (31.4)
ADHD	11 (29.7)
Depression	8 (21.6)
Cooccurring disorders	13 (35.1)
Other or unknown	5 (13.5)
Current mental health treatment	29 (78.4)
Prior psychiatric hospitalization	9 (24.3)
Suicide-related issues	
History suicide attempts	14 (11.9)
History of suicidal ideation	28 (23.7)
History of making suicidal statements or death wishes	30 (25.4)
Exposure to suicide	12 (10.2)
**Trauma**	
Suspected or confirmed abuse and/or neglect	20 (16.9)
Domestic violence	8 (6.8)
Death of a family member or friend	4 (3.4)
**Family-related problems**	
Divorce/custody issues	12 (10.2)
Legal problems	14 (11.9)
Parental substance abuse	8 (6.8)
Family history of psychological problems	7 (5.9)
Family history of suicide	6 (5.1)
**School/peer related problems**	
Recent expulsion/suspension	6 (5.1)
Recently changed schools	8 (6.8)
Special educational needs	7 (5.9)
Suspected or confirmed bullying	21 (17.8)

Abbreviation: ADHD, attention-deficit/hyperactivity disorder.

^a^ Precipitating circumstance known for 118 out of 134 cases.

**Figure.**
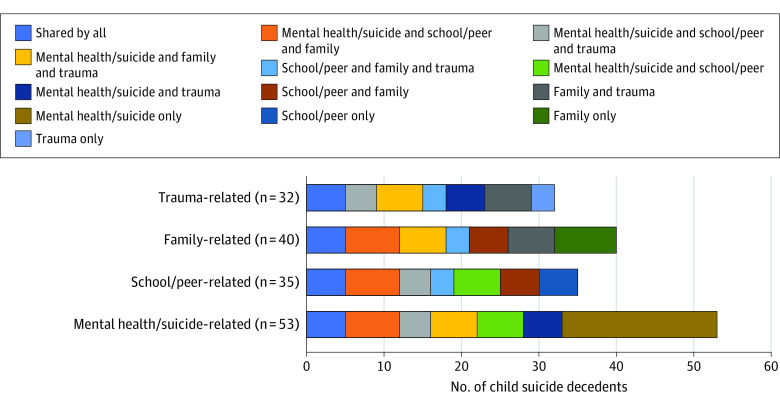
Themes Associated With Precipitating Circumstances From Coroner, Medical Examiner, and Law Enforcement Narratives of Suicide Decedents Aged 5-11 Years in 37 States and the District of Columbia, 2013-2017 The figure shows the 4 themes of precipitating circumstances for child suicide (mental health and suicide-related concerns, trauma, family-related problems, and school or peer-related problems) and the various thematic boundary overlaps found in this study.

### Mental Health and Suicide-Related Concerns

A mental health concern was specified for 31.4% (n = 37) of suicide decedents. ADHD, depression, and cooccurring disorders were the most common diagnoses. Among these children, 78.4% (n = 29) were receiving mental health treatment at time of death, and 24.3% (n = 9) had a prior psychiatric hospitalization. A history of suicide attempts and ideation was reported for 11.9% (n = 14) and 23.7% (n = 28) of children, respectively. One case stated, “the victim had a history of suicidal ideations since the age of five.” Previous suicidal statements and death wishes by decedents were noted in 25.4% (n = 30) of cases. In one example, parents received a call from school that the child was threatening to kill himself, and in another situation a child told her mother she was “better off dead,” and that “she should kill herself” in the week prior to her death. Child decedents commonly communicated suicidal statements and death wishes to peers. In one case, a friend told officials the decedent was “upset over a girl at school and said he was going to kill himself. She did not take him seriously because he had said things like that in the past.”

In 10.2% (n = 12) of cases the child had previous exposure to suicide. One case noted that the child was present when a grandmother attempted suicide, while another child had a schoolmate who died by suicide several weeks prior to the child’s death. Technology and the internet also played a role in exposing children to suicide. In one example, “the victim was observed by his mother playing suicide games on his electronic tablet” and in another “the victim’s principal learned from students there was a suicide challenge on social media”.

### Trauma

Reports of trauma occurred in 27.1% (n = 32) of cases and included suspected or confirmed abuse or neglect, domestic violence, and death of a family member or friend. In 40.6% (n = 13) of these cases, children experienced multiple traumatic events. In one situation, “the child was the victim of domestic violence, and saw his mother battle substance abuse. The victim’s mother overdosed and died in the weeks prior to the child’s suicide.” Another case reported “during the first two years of his life, the child was removed from his biological family numerous times due to issues of domestic violence, mental health, and abuse.”

### Family-Related Problems

One or more family-related circumstances including divorce and custody issues, other legal problems, parental substance abuse, and family history of psychological problems or suicide were documented in 39.8% (n = 47) of cases. In more than half of these cases (59.6% [n = 28]), these circumstances resulted in children living in single-parent households, blended families, or with other relatives. In one example, the child lived with his grandparents “who took custody of the child 6 years prior because their mother had severe mental health issues. The child has not seen his mother in 2-3 years, and never knew his biological father.” In another case the child resided with his mother’s boyfriend and children, because “his mother left approximately 4 years ago and is on the run, possibly for drug warrants. The child’s father is incarcerated.”

### School or Peer-Related Problems

School and peer-related problems were found in 35.6% (n = 42) of cases and included expulsion or suspension, a recent change in schools, or history of special educational needs. Suspected or confirmed bullying was identified in 17.8% of children (n = 21). Although bullying was more prevalent than any other reported school or peer-related problem, bullying alone did not appear to be a contributing factor. Among children who experienced bullying, multiple known risk factors for suicide^22,23^ cooccurred, including mental health problems and a history of suicidal thoughts and behavior. One case reported “the child was placed in a separate class due to behavioral issues and was being bullied at school. He had several mental health issues throughout his life. The child recently mentioned suicide to his school counselor.”

### On the Day of Suicide

Children were disciplined on the day of suicide in 32% (n = 38) of cases. Of these cases, school-related issues (34.2% [n = 13]) and an argument between child and parent or guardian (39.5% [n = 15]) preceded the disciplinary action. The most common punishment involved the child being sent to their bedroom (47.4% [n = 18]) and/or having their technological device taken away (28.9% [n = 11]). One example noted, “the decedent argued with his mother over poor grades. He was sent to his room and his iPad was taken away. The mother found him hanging from his bunk bed. The decedent had a history of ADHD, depression, and suicidal behavior.” A suicidal statement or death wish on the day of suicide was made by 11% (n = 4) of decedents. In one case, “text messages were discovered on the child’s phone the day of suicide stating she intended to kill herself.” In 76.9% (n = 29) of these children, similar remarks were made in the past. One situation noted, “the child told a friend on the day of death he was going to kill himself. Victim made suicidal statements before but never acted on them.” In 58.4% (n = 22) of cases it was reported that an adult was present in the home at the time of suicide.

## Discussion

Research on childhood suicide is limited, impeding our ability to develop critical prevention strategies. This study uniquely provides an in-depth examination of circumstances surrounding suicide in young children through a qualitative analysis of autopsy and law enforcement reports from multiple US states. This study’s results indicate that suicide in children is most often associated with numerous risk factors accumulated over time, including mental health concerns, prior suicidal behavior, trauma, and peer-, school-, or family-related problems. These findings further suggest a progression toward suicidal behavior, especially for youth with a history of psychopathology and suicidal behavior. Suicides were commonly preceded by a negative or precipitating event on the day of death, such as an argument between the child and a family member and/or disciplinary action.

Consistent with prior research,^15,22,23^ most child decedents were older, White, and male. Although suicide rates are traditionally higher in White than Black youth, racial disparities in childhood suicide rates have been identified.^14,24^ The current study revealed most children died by hanging or suffocation in their bedroom. The second most frequent method of suicide in children was by firearm. In all cases, the firearm was stored unsafely in the child’s home, underscoring the known association between firearm accessibility and suicide.^25,26,27^

School or peer-related issues were documented in more than one-third of cases. Suspected or confirmed bullying was reported more than any other school or peer-related problem, and in most cases overlapping risks for suicide such as mental health conditions and prior suicidal behavior were noted. These findings align with previous research suggesting the correlation between bullying and suicidal behavior is often mediated by other factors.^29,30,31^ One study in youth aged 6 to 18 years found bullying was significantly linked to suicidal behavior; however, this association was attenuated after controlling for depression and conduct problems.^31^

Problematic family circumstances were also commonly noted. Child decedents were often living apart from one or both parents, consistent with research suggesting youth with a history of suicidal behavior are more likely to be separated from their parents by divorce,^32^ family discord,^33,34,35^ or other adverse situations.^36,37^ Although only 11% of cases reported a family history of psychological issues or suicidal behavior, strong evidence suggests suicidal behavior is familial.^38,39,40^ Brent et al^39^ found offspring of parents with a history of suicide attempts were 6 times more likely to attempt suicide compared with offspring with no parental history.

Mental health concerns were identified in roughly 30% of cases, and 78% of these decedents were receiving mental health treatment. Similar to existing evidence, behavioral and mood disorders were the most frequent diagnoses.^6,7,15^ Approximately 12% of decedents reported a prior suicide attempt, which is similar to rates noted in previous studies.^41,42,43^ Decedents often had a history of suicidal ideation and/or making suicidal statements or death wishes, and expressed these comments on the day of suicide, suggesting suicidal statements should be taken seriously in younger children. Several decedents experienced prior exposure to suicidal behavior, and 1 in 4 had a history of trauma.

### Public Health and Clinical Implications

Strategies to prevent youth suicide have historically neglected to differentiate between children and adolescents.^44,45^ Aligned with a growing body of childhood suicide research,^3,4,5,6,7,14,15^ study results suggest investing in more effective suicide risk detection and targeted prevention initiatives for young children is essential. Research also indicates children who attempted suicide are up to 6 times more likely to attempt suicide in adolescence, offering further support for prevention efforts to begin with younger children.^46^

In our study, all children who died by firearm obtained a gun stored unsafely in the home. Household gun ownership is associated with higher rates of youth suicide,^25,26^ and evidence indicates safe storage practices can protect against unintentional firearm shootings and suicide attempts.^27,28^ Even modest improvements in firearm storage practices may prevent up to 32% of youth firearm deaths.^28^ Our findings underscore the importance of lethal means restriction interventions, including educational programs,^47^ youth focused firearm laws,^48^ and safe firearm storage public awareness campaigns.^47^

Strengthening relations between parents or caregivers and children can act as a protective factor for youth suicidal behavior.^8,9,32,33,34,35^ Family dissonance and instability was a common theme, suggesting family-based interventions could be impactful in preventing childhood suicidal thoughts and behaviors.^50,51,52,53,54^ One program, the Family Intervention for Suicide Prevention (FISP)/Safety-Acute(A), is a cognitive behavioral intervention for youth and parents or caregivers designed to decrease the risk of youth suicidal ideation and behavior.^52^ Attachment-Based Family Therapy (ABFT)^53^ and the Family-Based Crisis Intervention (FBCI)^54^ are additional empirically supported family therapy models designed to improve interpersonal family relations associated with depression and the risk for suicide.

Although close to one-third of suicide decedents had a documented mental health diagnosis, psychological autopsy studies report more than 90% of youth suicide decedents have a mental health condition,^55^ suggesting more robust mental health screening and suicide risk assessment is needed. Universal routine screening paired with lethal means safety counseling in pediatric primary care settings is recommended by suicide prevention researchers and the American Academy of Pediatrics to better detect at-risk youth.^56,57,58,59^ Screening children during a primary care visit can also inform parents about potential problems requiring intervention and facilitate referrals to specialty mental health services.^56,57,58^

Our finding that ADHD was the most common mental health diagnosis is particularly important given research showing childhood-diagnosed ADHD is a significant risk factor for future suicidal behavior.^60,61,62,63^ One study found children aged 4 to 6 years with ADHD were 3.6 times more likely to attempt suicide through the age of 18 years relative to children without a diagnosis.^61^

Standard treatments for ADHD in children include medication and behavioral therapy.^64^ A behavioral management program for children aged younger than 12 years shown to effectively reduce ADHD symptoms^62,63^ is the Incredible Years (IY) intervention.^65,66^ Although lacking demonstrated effectiveness in specifically reducing suicidal thoughts and behavior, IY addresses important developmental components critical to youth suicide prevention strategies.^67^

In 24% of cases, decedents experienced at least 1 traumatic event, which supports research linking childhood trauma and youth suicidal behavior.^68,69,70,71^ A meta-analysis found experiences of childhood maltreatment were associated with 2.5 to 4.0 greater odds for suicidal behavior compared with control groups.^68^ Taken together, findings suggest a trauma-informed approach toward youth suicide prevention may be warranted. An evidence-based intervention for children exposed to trauma is Trauma-Focused Cognitive Behavioral Therapy (TF-CBT), which may provide a promising integrated treatment for youth with trauma and cooccurring suicidal behavior.^72,73^ TF-CBT uses cognitive-behavioral principles with demonstrated efficacy for reducing suicide attempts in adults,^74^ along with risk factors associated with suicide in youth.^75^

### Limitations

There are some limitations with this study. First, restricted use NVDRS data were only available for select states and, therefore, findings are not nationally representative. Second, although NVDRS narrative information offers rich details regarding precipitating circumstances of suicides, data quality for incident narratives from autopsy and law enforcement reports varies by state and incident, introducing possible bias in study results. In addition, content for these retrospective reports are provided by parents, family members, and others familiar with the decedent who may not be aware of all circumstances associated with the child’s suicide. Third, potential bias from the misclassification of suicides as other causes of death^76,77^ may underestimate our findings, which may be a relevant issue in cases involving younger children. Fourth, the study’s qualitative analysis cannot confer causality, and future research is needed to establish whether certain characteristics and circumstances are actual risk factors for childhood suicide. Lastly, families and clinicians should not be unduly alarmed by study results, because the reported precipitating circumstances also pertain to children who will never engage in suicidal behavior.

## Conclusions

Using NVDRS data, this qualitative study found that childhood suicide was characterized by an interplay of multiple risk factors, commonly preceded by a negative precipitating event on the day of death. These findings support the need for early developmentally appropriate prevention strategies focused on more robust suicide risk assessment, improving family relations, and lethal means restriction. Future research is needed to clarify the myriad aspects of childhood suicide, including examination of racial/ethnic and sex differences.
